# Supplementary material for: The Geometric Phase of Stock Trading
Source: PLoS One. 2016 Aug 24;11(8):e0161538. doi: 10.1371/journal.pone.0161538 (PMC4996487; doi:10.1371/journal.pone.0161538)
Supplement: S1 File — (PDF) [file pone.0161538.s001.pdf]

SUPPLEMENTARY MATERIAL
The geometric phase of stock trading

C. Altafini
Division of Automatic Control, Dept. of Electrical Engineering,
Linköping University, SE-58183, Linköping, Sweden.
email: claudio.altafini@liu.se

August 13, 2016

Continuous-time model. The continuous-time equivalent of (1)-(3) of the paper is the following system of ODEs:

$$\begin{aligned} \dot{y}(t) &= u(t) && \text{(S1)} \\ \dot{s}(t) &= ru(t) && \text{(S2)} \\ \dot{z}(t) &= -s(t)u(t). && \text{(S3)} \end{aligned}$$

The geometric interpretation of the result shown in Fig. 1(a) of the paper for the model (S1)-(S3) is that if we consider $x \in \mathcal{M} \subset \mathbb{R}^3$ and the projection to the shape space $\mathcal{S} \subset \mathbb{R}^2$

$$\pi : \mathcal{M} \to \mathcal{S}$$
$$x \mapsto \begin{bmatrix} y \\ s \end{bmatrix}$$

then, given $x(0)$, for each trajectory $\gamma : [0,t] \to \mathcal{S}$ $\exists$ a unique $x(t) \in \mathcal{M}$ such that for the solution of (S1)-(S3), $x(t) = \pi^{-1}(\pi(x(t))$, i.e., the geometric phase variable $z(t)$ corresponding to $\gamma(t)$ is unique. In particular, if $\Gamma : [0,T] \to \mathcal{S}$ is a closed shape curve enclosing an area Ω, then the geometric phase (or "holonomy", [2, 1]) of Γ is

$$z(T) = z(0) + \oint_\Gamma s\, dy$$

or, by Stokes theorem,

$$\begin{aligned} z(T) &= z(0) + \int_\Omega d(s\, dy) \\ &= z(0) + \int_\Omega ds\, dy = z(0) + \omega \end{aligned} \tag{S4}$$

where ω is the area of Ω. It follows that when the area encircled by the cyclic trajectory Γ in shape space is zero (i.e., $\omega = 0$), then the phase variable z must shows no net displacement at the end of the cycle.

Indeed in the system (S1)-(S3), a cyclic trajectory in $u(t)$ induces a zero-area cycle in shape space $\mathcal{S}$ and does not produce any motion on the z variable, see Fig. A.

In other words, the geometric phase described in the paper is a purely discrete-time phenomenon, with no continous-time counterpart. In order to obtain a similar effect in continuous-time, it is necessary to produce cyclic trajectories of non-zero area in the shape space (y, s).

This can be achieved for instance by introducing a delay in the dynamics of s, representing a latency time in the response of the stock quote to buy/sell orders. Such delays are plausible if we focus on the very fast time scales preferred by high frequency traders. If we replace (S2) with

$$\dot{s}(t) = ru(t - \tau) \tag{S5}$$

where $\tau > 0$ is a time delay, then a periodic u-trajectory induces a nonzero area in the plane $\mathcal{S}$, and a net motion in the z variable is accumulated each time a cyclic trajectory is accomplished, see Fig. B.

As in the discrete-time case, it is possible to include in the model a spread between the buy and sell prices. Considering a quote spread in the continuous-time model means replacing (S3) with

$$\dot{z}(t) = \begin{cases} -a(t)u(t) & \text{if } u(t) > 0 \\ -b(t)u(t) & \text{if } u(t) > 0 \end{cases} \tag{S6}$$

Since a spread reduces the profit margins, in the delay-free continuous-time model (i.e., eq. (S1), (S2) and (S6)), it is impossible to attain a positive cash flow out of cyclic operations only (unlike in the discrete-time case), see Fig. C.

This confirms the qualitatively different prediction given by discrete and continuous-time models. Notice in Fig. C how a (negative) geometric phase is produced in spite of the zero-area of the cycle in shape space. This is however a pathological behavior, due to the discontinuity in the ODE (S6), and comes at the price of loss of uniqueness of the solution of (S6) when $u(t)$ crosses 0.

When also a delay is added to the continuous-time model (i.e., eq. (S1), (S5) and (S6) are considered), then a positive cash flow is again possible, depending on the numerical values of the parameters r, τ, and q, see Fig. D.

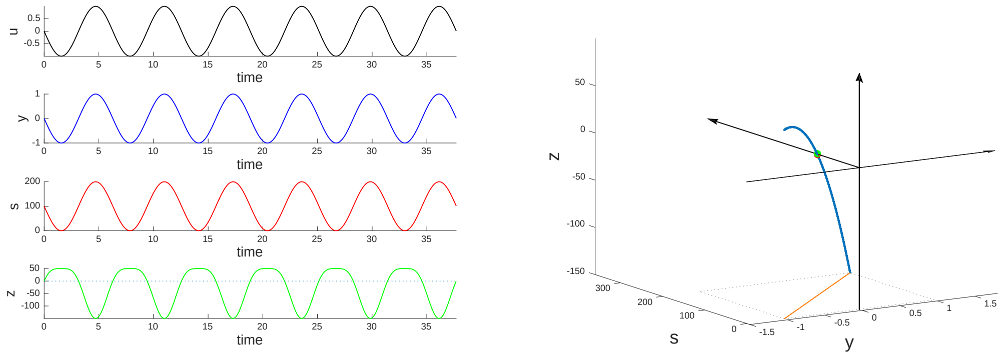

(a) (b)

Figure A: Continuous-time stock trading model: cycles of zero area in shape space (orange curve in panel (b)) yield no geometric phase. The starting point (green dot in panel (b)) and the final point (red dot in panel (b)) overlap.

References

[1] A. M. Bloch. *Nonholonomic Mechanics and Control*, volume 24 of *Interdisciplinary Applied Mathematics*. Springer-Verlag, 2003.

[2] J.E. Marsden and T.S. Ratiu. *Introduction to Mechanics and Symmetry*, volume 17 of *Texts in Applied Mathematics*. Springer-Verlag, 2nd edition, 1999.

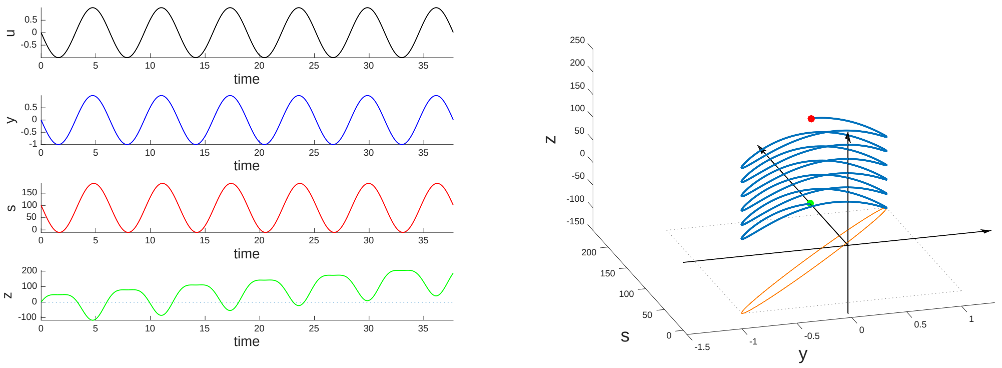

(a) (b)

Figure B: Continuous-time stock trading model: adding a time delay in one of the ODEs (here that for the stock quote s), is enough to induce a non-zero area in shape space and hence a geometric phase.

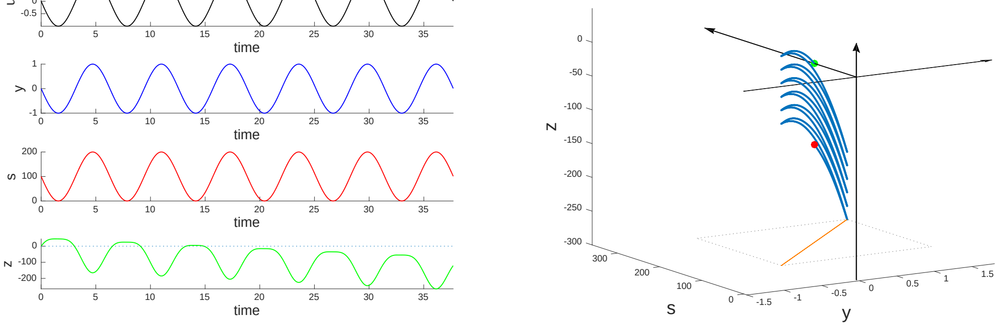

(a) (b)

Figure C: Continuous-time stock trading model with quote spread and no time delay. Even though the cycle has zero area in shape space, a geometric phase is induced but it necessarily corresponds to a loss. The resulting ODE for the cash balance is discontinuous and uniqueness of the solution is not guaranteed.

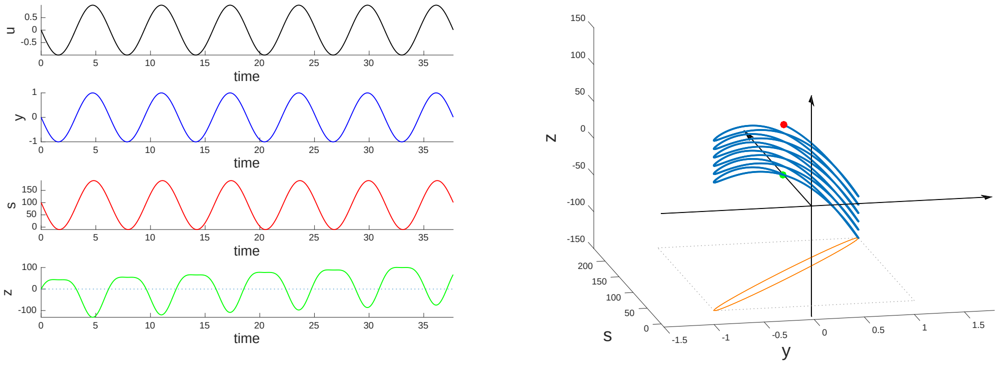

(a) (b)

Figure D: Continuous-time stock trading model: when both a time delay and a quote spread are considered, the geometric phase can correspond to a profit, depending on the values of r and q.
